# Plasmid transfer in biofilms: a perspective on limitations and opportunities

**DOI:** 10.1038/npjbiofilms.2016.22

**Published:** 2016-10-19

**Authors:** Thibault Stalder, Eva Top

**Affiliations:** 1 Department of Biological Sciences, University of Idaho, Moscow, ID, USA; 2 Institute for Bioinformatics and Evolutionary Studies, University of Idaho, Moscow, ID, USA

## Abstract

Biofilms dominate microbial life, and their importance for human health and the environment can no longer be dismissed. Nevertheless many of the processes governing this form of microbial growth are still poorly understood. This includes the horizontal exchange of genetic information, which is a major driver in bacterial evolution and rapid adaptation, exemplified by the alarming spread of multi-drug resistance among pathogens mediated by plasmids. Biofilms are often considered hot spot for horizontal gene transfer, yet several studies have shown that plasmid transfer is limited to the outer layers. On the basis of results from decades of research we analyse this paradox and discuss the mechanisms by which biofilm growth can promote the initial transfer of some plasmids, but also limit further plasmid invasion into the population or community. If we want to adequately promote or combat horizontal gene spread in biofilms, we need to gain better insight into the physicochemical and biological mechanisms that control this process.

## Introduction

Horizontal gene transfer (HGT) allows bacteria to rapidly adapt to changing environments, such as the presence of antibiotics, heavy metals or organic pollutants.^1^ One of the most important agents of HGT is plasmids, mobile genetic elements that replicate separately from the chromosome and can transfer to closely or distantly related bacteria.^2^ They increasingly make international news with alarming reports on their role in the spread of resistance to antibiotics of last resort.^3^ A wide variety of biotic and abiotic factors can affect the efficiency of plasmid transfer in and between bacterial populations. Examples are temperature, nutrient concentration, pH, moisture, population densities, cell signalling, cell physiology, type of plasmid, donor, or recipient, and growth on surfaces versus in well-mixed liquids.^4,5^ The latter parameter is of great importance as most bacteria in the environment, human microbiome and clinic live as biofilms, microcolonies or other forms of clumped cells with an explicit spatial structure.^6^ Moreover, some plasmids can promote biofilm formation by their bacterial host.^7^ Plasmid transfer has been shown to occur in many natural biofilm communities, such as soil, water, plant leaves, river rocks, biofilm reactors and mouse intestines.^8,9^ Conjugation events were observed at wildly varying frequencies, likely due to the diversity of parameters governing plasmid transfer. If we want to promote the spread of useful genes such as catabolic genes in bioremediation projects,^10^ or combat the spread of unwanted antibiotic resistance and virulence genes,^11^ we need to better understand the factors that affect gene transfer, in particular the role of biofilms. Specifically, we need to determine the effect of biofilm growth on (1) the frequency of initial gene transfer events and (2) the subsequent spread of plasmids through the community by horizontal or vertical transfer. Vertical plasmid transfer requires a plasmid to efficiently replicate in its host, and persist through strategies such as efficient segregation, post-segregational killing and minimising fitness cost.^12,13^ Here, we only focus on the horizontal transfer component of plasmid spread.

There is a general consensus in the literature that the stabilised cell-to-cell contact provided by biofilms promotes HGT by conjugation, as this mechanism requires cell contact. This has led to a quite generally accepted paradigm that biofilms promote HGT and can thus be considered HGT ‘hot spots’,^14–22^ previously defined as ecosystems, where plasmids transfer at high frequencies.^9^ This paradigm is often taken for granted and not always critically analysed. It is often interpreted as if the introduction of a few plasmid-bearing bacteria into a biofilm results in rampant plasmid invasion through horizontal spread, but several studies have shown this not to be the case.^23–30^ In this perspective, we focus on horizontal transfer of plasmids by conjugation in bacterial biofilms and other spatially structured populations, which have a central role in human and environmental health.

## Biofilms: heterogeneous and structured populations

A biofilm is broadly considered as a population or community of microorganisms grown on a surface or interface and embedded in a matrix of extracellular polymeric substances.^6,31^ Beyond this, there are no boundaries to the definition, leaving it up to us scientists to decide what can or cannot be considered a biofilm. For example, what is the minimum thickness of a biofilm, which affects the gradients of dissolved and/or gaseous nutrients, electron acceptors and waste products; and should biofilms be subjected to a continuous flow of nutrients? If microbial flocs (suspended bacterial aggregates) and bacteria grown on agar plates are judged to be biofilms, it widens the definition of a biofilm considerably.

One major feature of biofilms common to all interpretations of its definition is their heterogeneous character that can be structural, chemical, physiological and genetic in nature. This heterogeneity is generated by the spatial structure inherent to populations that are not continuously mixed. It profoundly affects how populations share genetic and physicochemical information. Moreover, the structures formed by multiple cell layers generate gradients of nutrients and gases. As a consequence, some individuals are actively growing, whereas others are in stationary or quiescent, or intermediate stages.^32^

## Spatial structure: a barrier for horizontal plasmid spread

Conjugative plasmid transfer has traditionally been studied in well-mixed broth or on agar plates, conditions that do not generally reflect biofilms. Well-mixed liquid cultures represent homogenous environments where individuals in the unstructured planktonic population randomly encounter each other and undergo multiple new encounters in a short time period, a feature that greatly affects plasmid transfer^5^ yet is highly unnatural. Like in biofilms, bacteria grown on agar surfaces represent heterogeneous, spatially structured populations, where cells are more or less fixed in space. However, typically the colonies receive nutrients from the agar below while gas exchange occurs at the top, and they are often very compact. In many biofilms grown on inert surfaces such as in flow cells, both liquid and gas exchange occur at the top, and there is more extracellular polymeric substance production than on agar plates. The differences in physiology of bacteria growing on agar and in biofilms under continuous flow have been previously discussed.^33^ It is easy to imagine that they differentially affect expression of transfer genes and thus plasmid transfer.

Several studies have compared the transfer efficiencies of plasmids in bacterial populations of donor and recipients cells that were initially well mixed and subsequently grown either as planktonic populations or on agar surfaces. In the early 80s, Bradley *et al.*^34^ found that for several broad-host-range plasmids with ‘short rigid pili’ the transfer frequency on an agar surface was 2,000- to 36,000-fold higher than in broth (more thoroughly reviewed by Frost^35^). Surface preference for mating was also reported for the *Pseudomonas putida* TOL plasmid.^36^ These studies demonstrate that close cell-to-cell contact and stabilised mating pair formation can improve the efficiency of conjugative transfer of some plasmids. They are often the most referenced in the literature to portray biofilms as hot spots for plasmid transfer.

**Figure 1:**
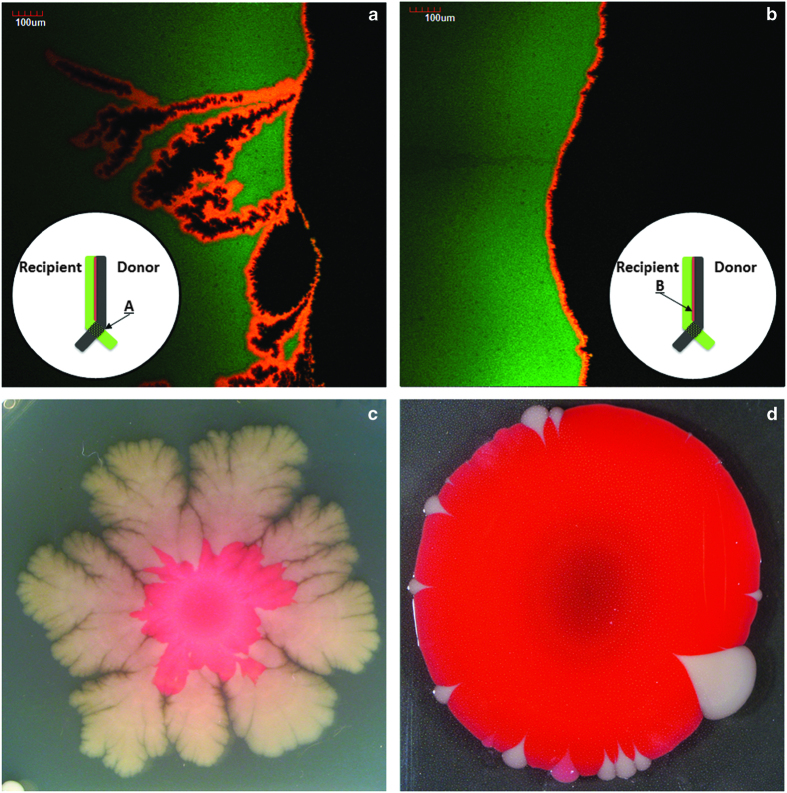
Plasmid transfer and loss in bacterial populations grown on agar. (**a**, **b**) Confocal laser scanning microscopy photographs of populations of donors of plasmid pB10 marked with *dsRed* (pB10::rfp in *P. putida* SM1443) and recipient cells (*P. putida* KT2442::gfp, green) grown at 30 °C for 4 days. Because the plasmid-borne *dsRed* expression is chromosomally repressed in the donor, the donor cells are black in the photograph. The bacteria were either streaked on lysogeny broth (LB) agar (**a**) in a cross, allowing initial mixing, or (**b**) next to each other (see schematics in the inserts for set-up); (**c**, **d**) photographs of two bacterial species forming colonies with sectors. The bacterial inoculum contained pB10::rfp and was allowed to grow for many days without selective pressure for plasmid retention. Loss of plasmid is visible as white sectors. All images support the notion that self-transmissible plasmids do not readily invade existing populations of plasmid-free cells.

In contrast, many studies have reported limited plasmid spread in spatially structured bacterial populations that are initially not well-mixed. For example, in a pioneering experiment Christensen *et al.*^37^ showed that conjugation occurred only during the early stages of contact between separate colonies of plasmid donors and recipients in clearly defined narrow zones wherein transfer frequencies were high (a similar example is presented in Figure 1a,b). However, further horizontal plasmid spread throughout the colonies was not observed. Several groups observed similar phenomena for other plasmids.^25,29,38,39^ In addition, colony growth of a plasmid-bearing population can result in clonal sectors of plasmid-free cells, with no apparent successful reinfection of plasmid-free cells by HGT^37,38,40^ (Figure 1c,d).

**Figure 2:**
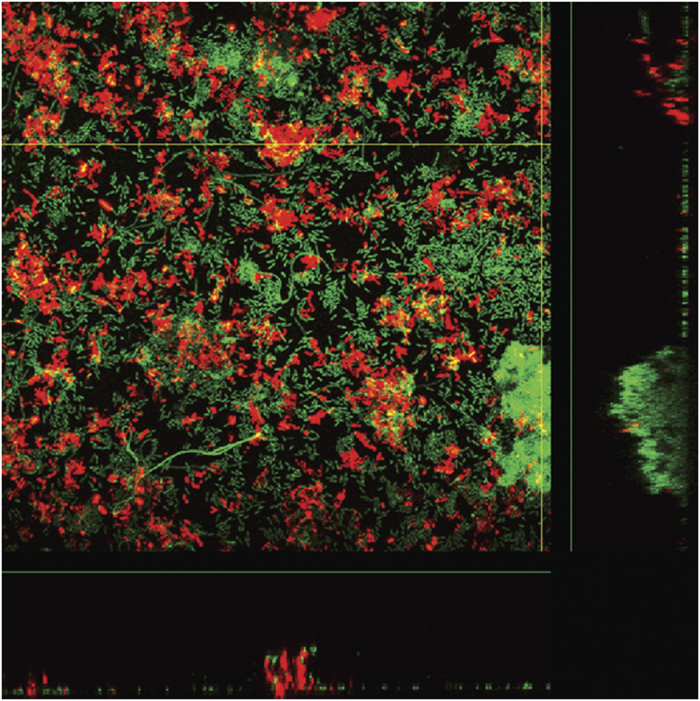
Confocal laser scanning microscopy photographs of a biofilm formed in a continuous flow chamber by a mixture of donor cells carrying plasmid pB10 marked with *dsRed* (pB10::rfp in *E. coli* MG1655, red) and recipient cells marked with *gfp* (*P. putida* KT2244::gfp, green), generating yellow/orange transconjugants at a low frequency. The mixture (ratio 1/1) of the two bacteria was grown for 27 h in a flow cell continuously fed with 1/3 M9.

*In situ* surveys of plasmid transfer in biofilms grown in flow cells have also shown that plasmid invasion in an established biofilm was detected only at the interfaces, where bacteria were most metabolically active and dividing.^23,25,26^ Licht *et al.*^27^ showed that plasmid invasion was proficient in a biofilm during the initial contact phase, after which no further transfer was detected. In contrast, the efficiency of invasion was initially slower in mixed liquids but constant, and eventually allowed plasmid spread through the entire population. The same authors found that consistent with a biofilm, plasmid invasion was also limited in a mouse gut model. Plasmid transfer from transconjugants, newly converted to donors, seemed limited rather than invasive (Figure 2). Earlier theory predicted that plasmids would be able to invade the entire colony by horizontal transfer as a wavefront because plasmid-bearing cells would always be in contact with recipient cells.^5,27^ So what is preventing this plasmid invasion?

## Factors that affect plasmid invasion in surface-grown bacterial populations

**Figure 3:**
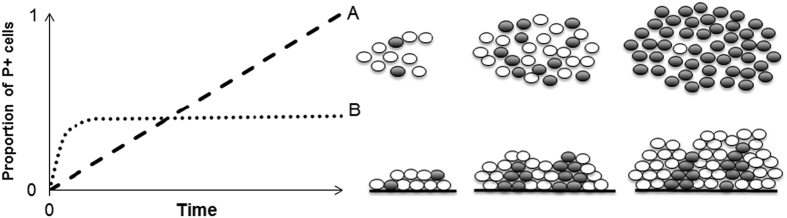
Theoretical representation of a self-transmissible plasmid that completely invades an unstructured population over time (A, dashed line) yet only partially invades a structured population (B, dotted line). The drawing on the right represents a population of plasmid-free (white) and plasmid-containing (dark) bacteria growing in a well-mixed environment (top) or on a surface (bottom). As discussed in the text plasmid invasion can occur by vertical and horizontal transfer.

The limited horizontal transfer of plasmids in spatially structured populations has been explained by multiple mechanisms. One of them is the physical isolation of distinct genotypes within large populations^41^ and the eventual isolation of donor and recipient cells. In a structured environment individuals only interact with their closest neighbours, thus creating subpopulations that are more or less independent from each other. This creates fewer opportunities for conjugative transfer. Fox *et al.*^24^ addressed this specific question using a joint experimental–theoretical approach with *Escherichia coli* on agar surfaces and a broad-host-range plasmid. The plasmid-containing cell fraction increased markedly over 2 weeks by HGT when nutrients were regularly replenished, but almost complete invasion only occurred when the structured population was disturbed daily, allowing new cell–cell encounters and thus new plasmid transfer events. This was consistent with the findings of Licht *et al.*^27^ described above, as a plasmid that transfers well in liquids invaded a well-mixed population due to the recurrence of new cell encounters. This concept is illustrated in Figure 3.

For several plasmid types the need for environmental or cell–cell signals to induce transfer competence in the donors has also been underlined as a potential cause of the limited spread of plasmids. As reviewed by Koraimann and Wagner,^42^ cells containing a conjugative plasmid are not necessarily able to transfer it by conjugation. Such regulatory control of the conjugation process can thus limit plasmid invasion. The transfer genes of some plasmids are downregulated in most actively growing cells, with only a fraction of cells able to activate their transfer genes. This so-called fertility inhibition allows bacteria to control the fitness cost associated with conjugation.^42^ However, fertility inhibition is inactivated when a plasmid first enters a new cell until sufficient repressor protein has accumulated, leading to a burst in transfer gene expression.^29,43^ Taking this phenomenon into account, Simonsen^5^ proposed that when donor and recipient colonies meet on a surface, the transitory derepression of conjugation could generate a frontal wave that sweeps through the recipient colony at high frequency. However, besides one report of infectious spread of a plasmid on an agar surface^44^ we are not aware of any studies that have observed such waves of plasmid transfer within surface-grown colonies or biofilms. There must be other factors preventing plasmid sweeps.^39^

The nature of the signals that trigger the switching between conjugation-deficient and -proficient cells in Gram-negative bacteria remain to be elucidated, especially in complex matrices such as biofilms. However, a few regulatory mechanisms in response to environmental and physiological cues such as quorum sensing, the SOS response, extracytoplasmic stress and gene silencing by histone-like nucleoid structuring protein (H-NS) have been shown to affect conjugative transfer of some plasmids.^45–49^ As cells in the deeper layers of thick biofilms are growing extremely slowly or not at all, a multitude of regulators that are at work there could very well limit conjugation.

The lack of plasmid invasion can also be explained by the lack of nutrients away from the growth edge of established donor and recipient colonies or biofilms. In the experiment of Fox *et al.*,^24^ no plasmid invasion was observed when nutrients were not replenished daily. Several studies including Seoane *et al.*^29^ found that conjugation depended on nutrient concentration, whereas others^50^ did not observe such nutrient dependence. As plasmid transfer and establishment in the recipient is a costly process,^51^ and typically only the edges of growing micro-colonies have access to an energy source, it follows that plasmids can rarely penetrate such clusters of cells.

Finally, factors such as oxygen availability, cell density, juxtaposition and cell-to-cell contact mechanics were shown to affect plasmid transfer in biofilms. For aerobic bacteria, high densities of well-mixed donor and recipient populations, often found at the liquid–air interface, are critical for successful plasmid spread.^26^ In addition, an *in silico* and experimental model at the individual cell level showed that cell elongation during growth facilitates conjugative plasmid transfer, which may thus be limited for spatially constrained cells in the centre of biofilms.^27,52^ This correlation between cell growth and conjugation has been also observed *in situ* in biofilms and on surface-grown colonies using fluorescent reporters,^25^ but the molecular mechanisms remain elusive.

## Conclusions and perspectives

To avoid confusion on whether or not biofilms promote plasmid-mediated HGT, we propose to make a clear distinction between the following three mechanisms: (1) the initial ability of plasmid-bearing cells to horizontally transfer their plasmid to neighbouring cells; (2) subsequent horizontal plasmid spread through the population or community; and (3) vertical plasmid spread. For the first step, whereas the signals that promote transfer competence are still poorly understood, biofilms are known to provide cell-to-cell contact and stabilise mating pair formation, thus increasing the likelihood of transfer for some plasmid types. However, these hot spots for conjugative transfer are restrained to subpopulations, small islands of donors and recipients that generate transconjugants but do not instigate a wave of horizontal plasmid spread through the biofilm. In contrast, biofilms appear to limit this second process, horizontal plasmid invasion, through a combination of physicochemical and biological factors inherent to the spatial structure and heterogeneity of biofilms. Whether a plasmid will then spread by vertical transfer entirely depends on the ability of the plasmid to persist in its new hosts, and of these hosts to locally outcompete their plasmid-free counterparts and other community members. The latter is in part determined by the selection on plasmid-encoded traits imposed by the biofilm environment. If we want to either promote the transfer of plasmids with useful genes, or conversely limit the spread of unwanted resistance or virulence genes, we need to get a better grip on the factors that affect each of these steps and thus jointly determine the fate of plasmids in biofilms.
